# Structural and Kinetic Properties of Liver Rhodanese from *Coptodon zillii*: Implications for Cyanide Detoxification in Gold Mining-Impacted Aquatic Ecosystems

**DOI:** 10.3390/toxics13090750

**Published:** 2025-09-03

**Authors:** Oluwaseun E. Agboola, Zainab A. Ayinla, Babamotemi O. Itakorode, Priscilla O. Akinsanya, Raphael E. Okonji, Othuke B. Odeghe, Samuel S. Agboola, Olaiya E. Oluranti, Folake O. Olojo, Babatunji E. Oyinloye

**Affiliations:** 1 Institute of Drug Research and Development, S.E Bogoro Center, Afe Babalola University, PMB 5454, Ado-Ekiti 360001, Nigeria; 2 Damsem Scientific Laboratory and Research, Ado-Ekiti 360102, Nigeria; 3 Department of Biochemistry and Molecular Biology, Obafemi Awolowo University, Ile-Ife 220103, Nigeria; itakorgsoli29@gmail.com (B.O.I.); priscilaakinsanya@gmail.com (P.O.A.);; 4 Department of Biology, University of Waterloo, Waterloo, ON N2L 3G1, Canada; zayinla@uwaterloo.ca; 5 Department of Biotechnology, Osun State University, Osogbo 210001, Nigeria; 6 Department of Medical Biochemistry, Faculty of Basic Medical Sciences, Delta State University, Abraka 320002, Nigeria; bensandym@yahoo.com; 7 Department of Pharmacology and Toxicology, Afe Babalola University, Ado-Ekiti 360102, Nigeria; agboolass@abuad.edu.ng; 8 Department of Medical Biochemistry, College of Medicine and Health Sciences, Afe Babalola University, Ado-Ekiti 360102, Nigeria; olaiyaoe@abuad.edu.ng; 9 Department of Chemical Sciences, Dominion University, Ibadan 110108, Nigeria; f.olojo@dominionuniversity.edu.ng; 10 Phytomedicine, Biochemical Toxicology and Biotechnology Research Laboratories, Department of Biochemistry, College of Sciences, Afe Babalola University, PMB 5454, Ado-Ekiti 360001, Nigeria; 11 Biotechnology and Structural Biology (BSB) Group, Department of Biochemistry and Microbiology, University of Zululand, KwaDlangezwa 3886, South Africa

**Keywords:** rhodanese, cyanide detoxification, *Coptodon zillii*, gold mining, environmental adaptation, bioremediation

## Abstract

The global gold extraction industry has been reported to use cyanide-based recovery processes, which pose environmental effects on water resources. The study examined *Coptodon zillii* liver rhodanese from a gold mining-impacted reservoir with a specific focus on the enzyme’s critical function in cyanide detoxification. Rhodanese was purified using successive chromatographic techniques with 5.4 U/mg specific activity and 3.1-fold purification. The molecular weight of the native enzyme was 36 kDa, and the subunits were 17 kDa, indicative of a dimeric structure. Optimal enzymatic activity was recorded at pH 8.0 and 50 °C. The effect of metal ions was significantly varied: the activity was inhibited by BaCl_2_, CaCl_2_, NaCl, and MgCl_2_, and KCl enhanced performance. The kinetic determinations showed Michaelis-Menten kinetics with a Km of 20.0 mM for sodium thiosulfate and 25.0 mM for potassium cyanide. The enzyme’s minimal activity was identified toward 2-mercaptoethanol, ammonium persulfate, and ammonium sulfate, but with evidence of preference for thiosulfate utilization under the substrate specificity tests. The major interactions between the enzyme and the substrate were revealed by the molecular docking experiments. These showed Glu159, Gln161, and Arg173 formed important hydrogen bonds with thiosulfate, while Arg156 and Val172 were also involved. Other substrates are bound to Gln121 and Trp139 residues with much lower binding energy than thiosulfate. The findings increase our understanding of biochemical adaptation process knowledge in anthropogenically stressed environments, showing strategies of ecological resilience. The characterized enzymatic features showed potent cyanide detoxification potential, and the possible applications are in bioremediation strategies for mining-impacted aquatic ecosystems.

## 1. Introduction

Food security is of global concern due to environmental constraints and population growth [1]. Over 9.7 billion people are projected to be on the planet by 2050; therefore, there will be more demand for cheap sources of protein [1]. Most of the world’s protein intake comes from fish, providing crucial nutrients to almost 3 billion people globally [2]. Besides nutritional benefits, fish is also a cheap source of protein, particularly in poor nations where other sources are still beyond reach [2]. Among all the types of fish, tilapia is the most significant aquaculture species worldwide, producing at least 6 million tonnes annually [3]. The secret to success is great biological traits like environmental tolerance, great feed efficiency, and fast growth [3]. Red-belly tilapia, or *Coptodon zillii*, find a growing leading position in African, Asian, and Latin American capture and aquaculture fisheries [4]. The species is highly morphologically adaptable and is found in various forms of aquatic habitats, like lakes and brackish coastal waters [4]. It is highly tolerant of diverse environmental conditions, and due to this fact, it has gained a central position as a food-security organism in developing nations [4]. However, the increased scale of gold mining activities is one of the largest hazards to water bodies inhabited by such fish. The gold mining international business has seen tremendous expansion, with jewelry, electronics, and investment segments being in the lead [5]. Existing gold mining activities are bound to utilize cyanide-based methods, and, thus, there is pollution of the environment [5]. Sodium cyanide and potassium cyanide use are applied for heap leaching and carbon-in-pulp activities and cause unplanned leaks, seepage of tailings ponds, and uncontrolled waste disposal [5]. The activity impacts nearby water bodies in and around mining sites in prominent nations known for gold production, including China, Australia, South Africa, and other regions in South America [5]. Cyanide exposure extends past acute toxicological concerns [5]. The chemicals are stable in aqueous medium under alkaline pH or low oxygen conditions and interfere with cell processes by binding to cytochrome c oxidase and inhibiting mitochondrial electron transport [6]. This induces cellular hypoxia, resulting in immediate death in sensitive organisms, but sublethal exposure results in chronic stress at the physiological level, slower growth rates, depressed reproduction, and increased vulnerability to disease [6]. Survival of aquatic organisms in cyanide-contaminated environments depends upon their capacity to detoxify [6]. These processes are an expression of evolutionary processes that evolved as a response to the naturally occurring cyanogenic compounds in plant organisms and microorganisms [6]. It makes use of the mitochondrial enzyme rhodanese in the process [6]. It detoxifies cyanide to less poisonous thiocyanate through the transfer of sulfur from thiosulfate donors; it is otherwise referred to as thiosulfate:cyanide sulfurtransferase [6]. It facilitates energy production and cellular respiration, which are central to the organic life of an organism [6]. The rhodanese enzyme family is varied across taxonomic groups, exhibiting variations in cellular localization, substrate specificity, and catalytic efficiency [6]. Because fish liver tissue contributes to the majority of xenobiotic metabolism, it should also display the maximum enzymatic activity [6]. The catalytic process of the enzyme involves passing through the covalent enzyme-sulfur intermediate, followed by sulfur transfer to the cyanide substrate to form readily excretable thiocyanate [6]. Even though there has been extensive work on cyanide toxicity in water, data on food fish species adaptation towards chronic cyanide exposure are still slim. Laboratory-based exposure methods with naive stocks of fish are the methods most toxicological studies employ, which cannot be applied in predicting adaptive response by environmentally stressed long-term exposed wild populations [5]. Previous research has been concerned with acute toxicity and has omitted to address the knowledge gaps as far as biochemical adaptive mechanisms to enable fish populations to survive where there is pollution [5]. The presence of *C. zillii* in gold mining-polluted water bodies implies the presence of adaptive coping mechanisms that are worth exploring [4]. Such populations would have likely complied with genetic or epigenetic changes that increase cyanide detoxifying capacity through enhanced rhodanese production, modified enzyme kinetics, or increased cofactor availability [6]. These adaptations can form the foundation of aquaculture development schemes in mine lands and guide environmental conservation schemes, harmonizing economic development and safeguards to ecosystems [4]. Clarification of the *C. zillii* cyanide tolerance molecular mechanism may disclose enzymatic properties beneficial for bioremediation technology [6]. Better rhodanese structures could be accountable for successful treatment methods for cyanide-poisoned water bodies that favor aquatic life and support human populations [5]. This study investigates the chemical properties and catalytic activity of *C. zillii* liver rhodanese from a gold mining-impacted reservoir. By extensive biochemical characterization through kinetic parameter determination and structure, our research clarifies certain adaptations enabling this valuable economic species to thrive in polluted ecosystems. The findings aspire to contribute to the understanding of the biochemical process of cyanide tolerance in aquaculture food fish and make sustainable aquaculture practice feasible in mining-polluted regions.

## 2. Materials and Methods

### 2.1. Materials, Sample Collection, and Authentication

All the chemicals and reagents used were of analytical grade. Sodium thiosulfate, potassium cyanide, bovine serum albumin (BSA), and phenylmethylsulfonyl fluoride (PMSF) were procured from Sigma-Aldrich (St. Louis, MO, USA). Tris-HCl buffer components were bought from Merck (Darmstadt, Germany). Spectrophotometric measurements were collected using a UV-Vis spectrophotometer (Shimadzu UV-1800, Kyoto, Japan). Protein electrophoresis was performed using Bio-Rad Mini-PROTEAN equipment (Hercules, CA, USA). Specimens of *Coptodon zillii* (n = 15) were taken at Igun Gold Mine Reservoir (004°30′ E–004°43′ E and 07°31′ N–07°35′ N) in Osun State, Nigeria, where regular mining activity occurs. The location exhibits cyanide levels of 0.08–0.12 mg/L, above WHO allowed limits (0.07 mg/L) for aquatic life protection. Only *C. zillii* was taken as the sole fish species present in the contaminated reservoir. The Department of Zoology, Obafemi Awolowo University, authenticated species identification employing established taxonomic keys [7].

### 2.2. Enzyme Extraction and Purification

Fresh liver tissue (45 g) pooled from all fifteen specimens was homogenized in 0.1 M phosphate buffer, pH 7.2 (1:3 *w*/*v*), containing 1 mM phenylmethylsulfonyl fluoride (PMSF) as a protease inhibitor [8]. The homogenate was centrifuged at 4000 rpm for 30 min at 4 °C using a Beckman J2-21 refrigerated centrifuge. Protein precipitation was employed with 70% ammonium sulfate. Ion-exchange chromatography using CM-Sephadex C-50 (1.5 × 10 cm column) equilibrated with 0.1 M Tris-HCl buffer (pH 7.2). The proteins were eluted with a 0.1 M NaCl gradient at a 40 mL/hour flow rate, collecting 2 mL fractions [9]. Active fractions underwent gel filtering on Biogel P-100 following standard methods.

### 2.3. Enzyme Assay and Protein Determination

Rhodanese activity was evaluated spectrophotometrically at 460 nm following thiocyanate production [10]. The reaction mixture (1.0 mL) contains 0.5 mL borate buffer (pH 9.4), 0.2 mL KCN (250 mM), 0.2 mL Na_2_S_2_O_3_ (250 mM), and 0.1 mL enzyme solution at 37 °C. One unit reflects the enzyme quantity converting 1 µmol cyanide to thiocyanate per minute. Protein concentration was determined using the Bradford method using BSA as a standard [11].

### 2.4. Molecular Weight Determination

SDS-PAGE (10% gel) assessed subunit molecular weight using the Bio-Rad Mini-PROTEAN system [12]. The standard proteins comprised β-galactosidase (118 kDa), bovine serum albumin (BSA) (60 kDa), ovalbumin (45 kDa), carbonic anhydrase (29 kDa), and trypsin inhibitor (20 kDa). A Sephadex G-100 column (2.5 × 90 cm) calibrated with BSA (60 kDa), ovalbumin (45 kDa), pepsin (35 kDa), and α-chymotrypsinogen (25 kDa) standards was used to determine the native molecular weight. Phosphate buffer (pH 7.2) was used to elute proteins at a rate of 20 milliliters per hour, collecting 5-milliliter fractions that were measured at 280 nm.

### 2.5. Kinetic Studies

Initial velocity tests employed different doses (5–50 mM) of KCN and Na_2_S_2_O_3_. Lineweaver–Burk plots determined the Km value [13].

### 2.6. pH and Temperature Studies

Buffer systems (50 mM) ranged from pH 5.0 to 11.0: citrate (5.0–6.0), phosphate (6.5–7.5), Tris-HCl (8.0–9.0), and borate (9.5–11.0). Temperature effects were examined between 30 and 100 °C using normal assay conditions. For stability experiments, enzyme solutions were pre-incubated at specified temperatures for 60 min, with sampling at 10 min intervals for residual activity measurement [8].

### 2.7. The Impact of Salts on Rhodanese Activity

The impact of different salts on the activity of the rhodanese enzyme that was isolated from the liver of *Coptodon zillii* was examined using the technique outlined by Lee et al. [10]. Using a standard rhodanese assay combination, 250 mM concentrations of BaCl_2_, CaCl_2_, KCl, NaCl, and MgCl_2_ were tested. The metallic chlorides dissolved in distilled water. The reaction mixture was used as a control with 100% activity in the absence of the salts.

### 2.8. Studies on Substrate Specificity

Rhodanese’s substrate specificity has been extensively investigated using a variety of sulfur-containing compounds. Equimolar concentrations (20 mM) of each chemical were assessed using standard assay procedures. Ammonium persulphate, ammonium sulphate, sodium thiosulphate (Na_2_S_2_O_3_), and 2-mercaptoethanol were among the compounds that were analyzed. 0.5 mL of borate buffer (pH 9.4), 0.2 mL of KCN (250 mM), 0.2 mL of sulfur compound (250 mM), and 0.1 mL of enzyme solution made up the reaction mixture (1.0 mL). Activity was measured at 37 °C using standard rhodanese test procedures, and absorbance was measured at 460 nm. Sodium thiosulphate was used as the reference substrate (100%), and the results were displayed as percentage relative activity [9].

### 2.9. Studies on Heat Stability

Temperatures between 30 °C and 60 °C were used in thermal stability studies. A temperature-controlled water bath (Techne Inc., Princeton, NJ, USA) was used to pre-incubate aliquots of pure enzyme in 50 mM borate buffer (pH 9.4) at different temperatures (30, 40, 50, and 60 °C). Samples (0.1 mL) were extracted at 10 min intervals over a 60 min period and tested right away for residual activity under standard conditions.

### 2.10. Measures for Quality Control

Calibration tests are conducted on a regular basis with validated reference materials. Every test was conducted with blank controls. International biochemistry guidelines were followed in the inclusion of appropriate negative and positive controls in enzyme experiments.

### 2.11. Computational Study

Computational experiments were performed utilizing the rhodanese domain-containing protein from *Oreochromis niloticus* (UniProt ID: A0A669B6V2) as a suitable homolog, due to the unavailability of the *Coptodon zillii* rhodanese structure. The 3D structure was derived from AlphaFold (AF-A0A669B6V2-F1-v4) and verified using Ramachandran plot analysis. The protein structure was built using AutoDock Tools 1.5.7, including the insertion of polar hydrogens and Kollman charges. The binding site was found using Discovery Studio Visualizer 2021, focused on the conserved catalytic pocket.

Ligand structures (sodium thiosulfate, 2-mercaptoethanol, ammonium persulfate, and ammonium sulfate) were improved using the MMFF94 force field [14,15,16]. AutoDock Vina was applied for molecular docking simulations with a grid box centered on the identified catalytic site (dimensions: X = −4.60 Å, Y = 1.47 Å, Z = 13.91 Å) [17,18,19,20,21]. Using Discovery Studio Visualizer, the best conformations were chosen based on binding energies, and protein-ligand interactions were investigated. A cutoff distance of 3.5 Å was used to characterize hydrogen bonds, hydrophobic interactions, and other non-bonded interactions.

### 2.12. Data Analysis

All experiments were performed in triplicate, expressing data as mean ± standard error (SE). Error bars in figures represent the standard error of the mean (n = 3). Statistical analysis was performed using GraphPad Prism 8.0, applying one-way ANOVA with Tukey’s post hoc test. Significance threshold set at *p* < 0.05.

## 3. Results

### 3.1. Enzyme Purification and Molecular Features

A methodical purification approach was used to successfully isolate rhodanese from the liver of *C. zillii*. Our first crude extract produced 690 total units from 401 mg of protein, and the first purification step employing 70% ammonium sulfate precipitation recovered 252 units, indicating a 1.63-fold increase in specific activity (Table 1).

**Figure 1:**
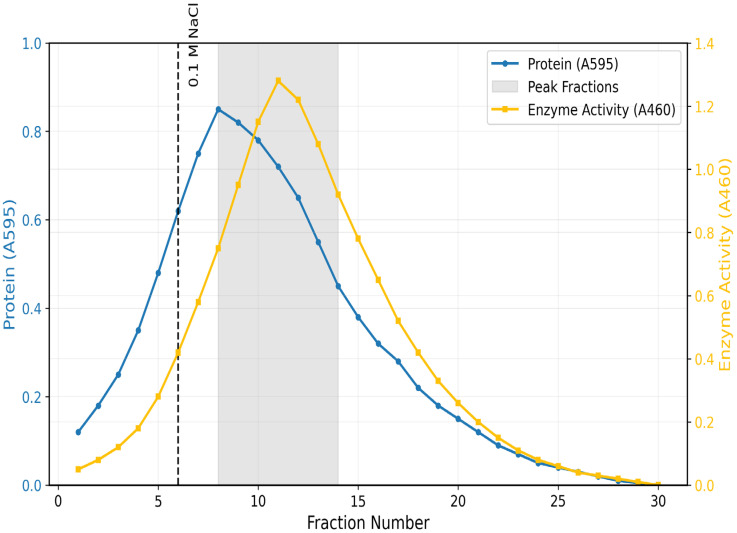
Elution Profile of *Coptodon zillii* Liver Rhodanese on CM Sephadex C-50 Ion Exchange Chromatography. 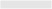 Pooled Fractions.

**Figure 2:**
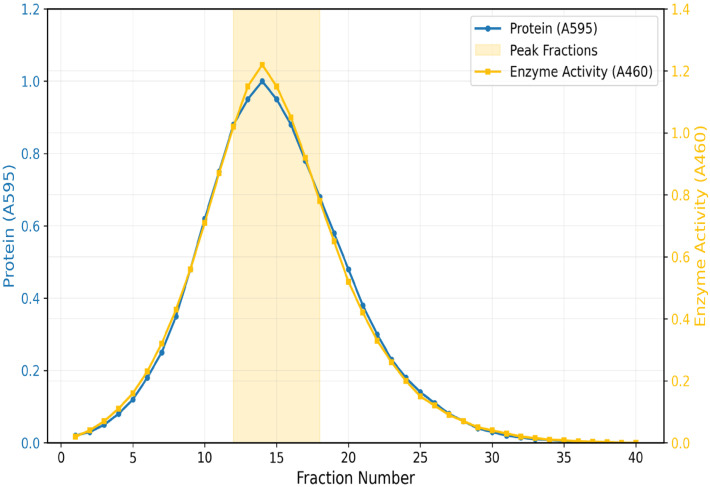
Elution Profile of *Coptodon zillii* Liver Rhodanese on Biogel P-100 Gel Filtration Chromatography. 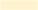 Pooled Fractions.

A typical elution profile was achieved by ion-exchange chromatography set on CM-Sephadex C-50. Active fractions under 0.1 M NaCl gradient elution were a symmetrical single peak (Figure 1). It retrieved 140.08 total units and improved specific activity up to 3.3 U/mg. For the final purification, gel filtration on Biogel P-100 was required (Figure 2). The enzyme eluted as a single peak under a molecular mass of 36 kDa. This last step achieved a 3.1-fold purification, 16% yield, and specific activity of 5.4 U/mg (Table 1).

### 3.2. Kinetic Properties and Substrate Interactions

Control tests demonstrated little non-enzymatic interaction between thiosulfate and cyanide under assay conditions, verifying the enzymatic specificity of reported catalysis. A kinetic study utilizing Lineweaver–Burk plots revealed unique substrate interactions. The enzyme displayed Km values of 20.0 mM and 25.0 mM for sodium thiosulphate and potassium cyanide, with Vmax values of 1.7 and 5.0 rhodanese units (RU), respectively.

**Figure 3:**
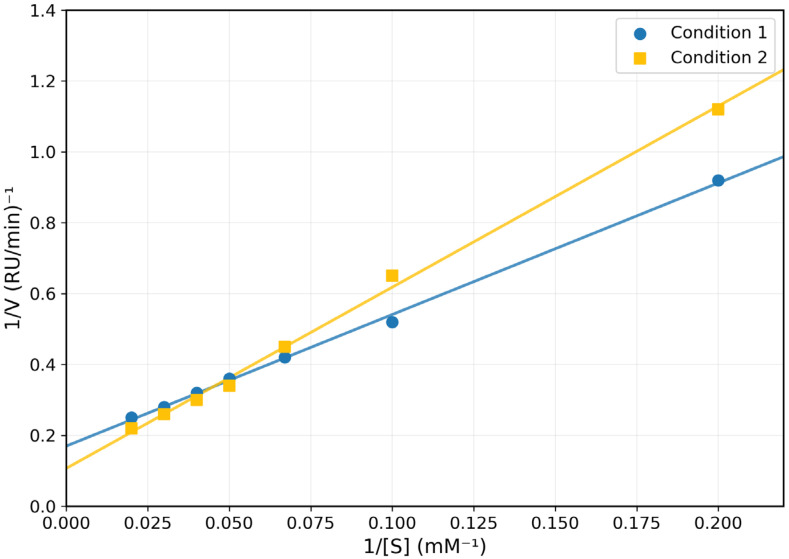
Lineweaver–Burk Plot for Varying Concentrations of Sodium Thiosulphate (Na_2_S_2_O_3_) (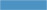) and Varying Concentrations of Potassium Cyanide (KCN) (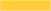) for *Coptodon zillii* Liver Rhodanese.

**Figure 4:**
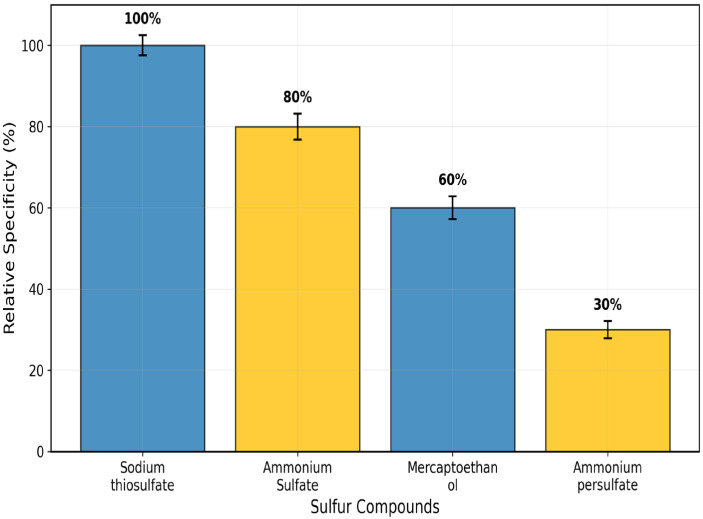
Seasonal Substrate Specificity Chart of some Sulphur-Containing Compounds. Values represent mean ± SE (n = 3).

In Figure 3, substrate specificity investigations corroborated this preference tendency. Among the studied sulfur compounds, relative activity followed the order: sodium thiosulphate (100 ± 2.5%) > ammonium sulphate (80 ± 3.2%) > 2-mercaptoethanol (60 ± 2.8%) > ammonium persulphate (30 ± 2.1%) (Figure 4).

### 3.3. Effect of pH, Temperature, and Salts

**Figure 5:**
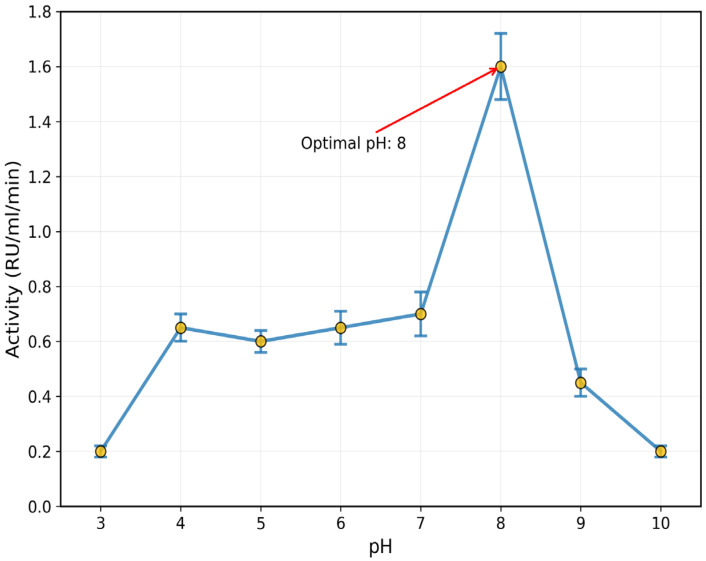
Effect of pH on *C. zillii* Liver rhodanese Activity. Data points show mean ± SE (n = 3).

**Figure 6:**
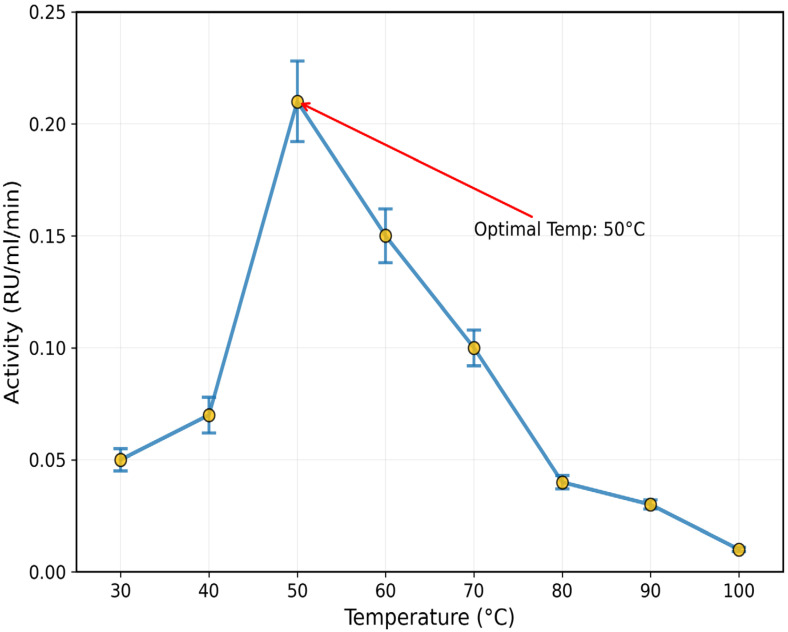
Effect of Temperature on the Activity of *C. zillii* Liver rhodanese. Data points show mean ± SE (n = 3).

**Figure 7:**
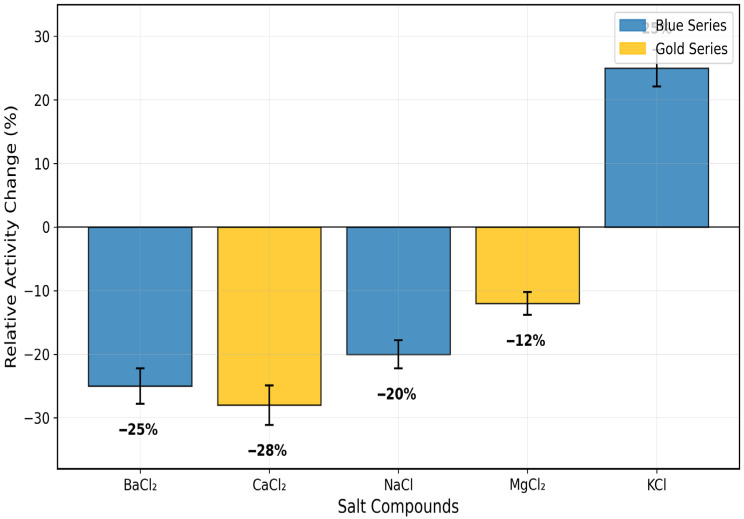
Effect of Salts on *Coptodon zillii* Liver Rhodanese Activity. Values represent mean ± SE (n = 3).

The purified enzyme recorded an optimum pH of 8 (1.6 ± 0.12 RU/mL/min) and temperature of 50 °C (0.21 ± 0.018 RU/mL/min) (Figure 5 and Figure 6). The inhibitory effect of some salts, such as BaCl_2_, CaCl_2_, NaCl, and MgCl_2_, was confirmed on the activity of rhodanese from the liver of *Coptodon zillii*, in the range of 12 ± 1.8% to 28 ± 3.1%, while KCl enhanced the enzyme activity by 25 ± 2.9% (Figure 7).

### 3.4. Thermal Stability Profile

**Figure 8:**
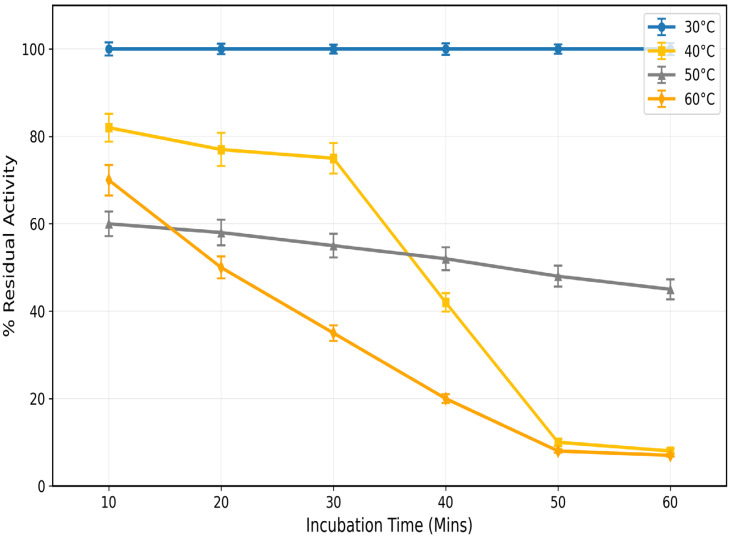
Thermal Stability of *Coptodon zillii* Liver Rhodanese. Data points show mean ± SE (n = 3).

Temperature studies revealed optimal stability at 50 °C. The enzyme retained 57% activity after 60 min at this temperature. Activity persistence followed a clear pattern:40 °C (57%) > 50 °C (46%) > 60 °C (44%) (Figure 8). Increased relative activity retention at 50 °C over 40 °C can be taken to indicate that the enzyme has maximum structural stability at the higher temperature, when it is in a more favorable energy conformation. The trend for temperature-dependent stability indicates that the optimum working range of the enzyme, permitting optimum active site geometry and substrate access, is in the vicinity of 50 °C. From the apparent difference, it is feasible to maintain enzymatic activity for 40 min of incubation by successfully altering the structure to counteract the 40 °C initial denaturation processes that result in lasting catalytic efficiency at 50 °C (Figure 8).

### 3.5. Structural Analysis

**Figure 9:**
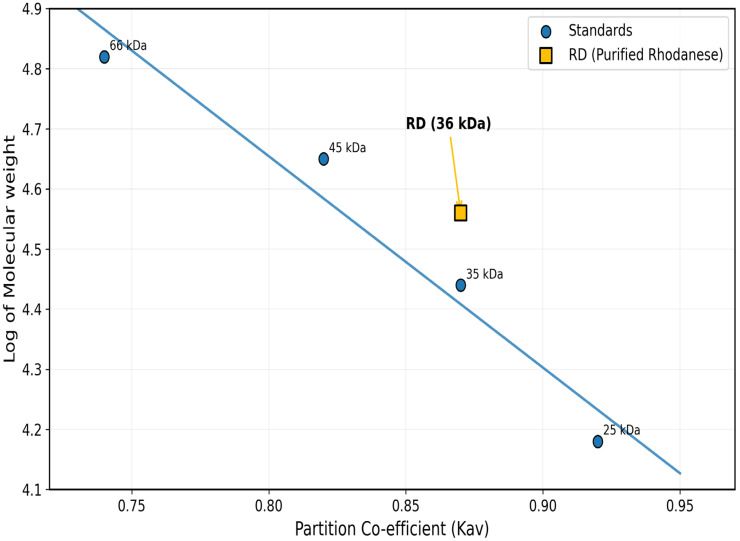
Native Molecular Weight Determination Standard Plot. BSA (66 kDa), ovalbumin (45 kDa), pepsin (35 kDa), and α-chymotrypsinogen (25 kDa) and RD: purified rhodanese (36 kDa).

**Figure 10:**
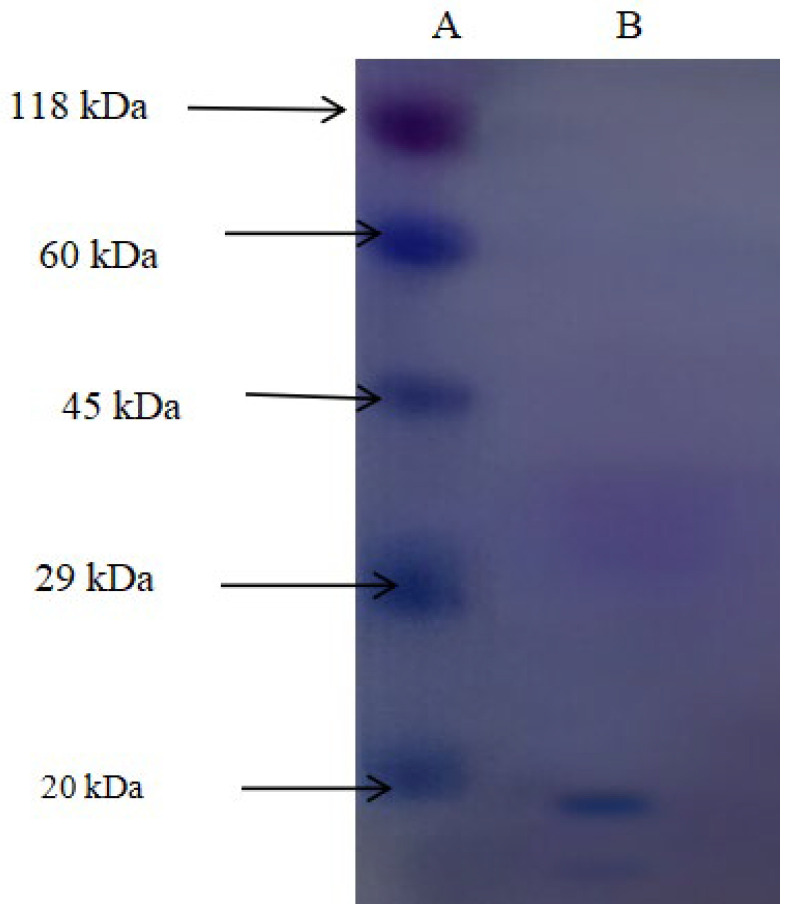
SDS PAGE Profile of the Purified Rhodanese. Lane A: Standard proteins: β-galactosidase (118 kDa), BSA (60 kDa), ovalbumin (45 kDa), carbonic anhydrase (29 kDa), and trypsin inhibitor (20 kDa), while Lane B = purified rhodanese (17 kDa).

A native molecular weight of 36 kDa was confirmed on the gel filtration. Molecular mass and homogeneity of the enzyme were established by SDS-PAGE electrophoresis. SDS-PAGE analysis revealed a single protein band of 17 kDa (Figure 9 and Figure 10). These observations indicate that the enzyme occurs as a homodimer in the native condition, which is consistent with the conventional quaternary structure found in rhodanese enzymes from other species. All purification and characterization parameters confirm that a detoxifying enzyme for cyanide is highly appropriate. The kinetic parameters and the stability properties are accurately within the environmental conditions found in the natural environment of *C. zillii*.

### 3.6. Outcomes of Molecular Docking

**Figure 11:**
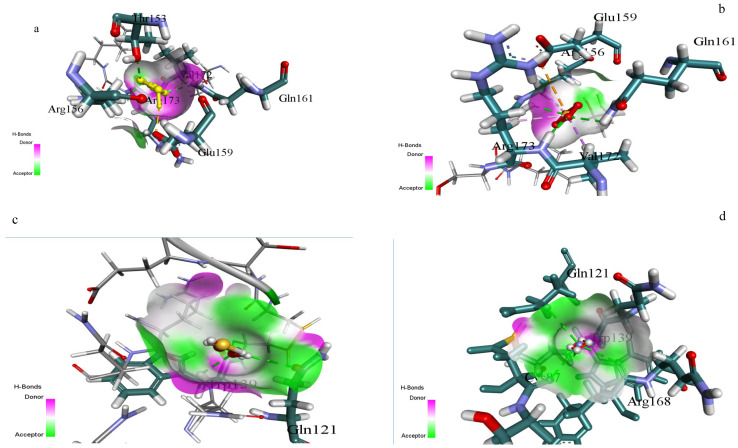
Molecular Interactions of four different Substrates with Rhodanese Enzyme. (**a**): Sodium thiosulphate Interaction (**b**): Ammonium Sulphate Interaction (**c**): Mercaptoethanol Interaction (**d**): Ammonium Persulphate Interaction.

The different substrate binding modes were established in the active site pocket of rhodanese using molecular docking studies (Table 2 and Figure 11). Sodium thiosulfate established significant interactions with Glu159, Gln161, and Arg173 residues, while essential stabilization was contributed by Arg156 and Val172. Ammonium persulfate and 2-mercaptoethanol established weak interactions with Gln121 and Trp139, while other sulfur compounds displayed different orientations of binding. Substrate orientation analysis revealed an unmistakable thiosulfate preference through sustained patterns of interaction and binding energies. The enzyme’s thiosulfate selectivity as the major sulfur donor was proved through the conserved residues, which constructed a typical catalytic pocket. The thiosulfate specificity of the enzyme was also established on the basis of differences in surface interactions, interacting residues, and hydrogen bond donors and acceptors among the substrates (Table 2 and Figure 11).

## 4. Discussion

The industrial contamination of aquatic ecosystems by artisanal miners presents unheard challenges to global food security [22]. Cyanide and associated toxins are introduced into watersheds that are essential for the production of protein by mining operations, especially in developing nations [23]. Promising biotechnological solutions for sustainable aquaculture in compromised environments are provided by the characterization of effective detoxification systems from hardy species like *C. zillii*.

This is the full description of *Coptodon zillii* (previously *Tilapia zillii*) rhodanese. It gives novel information on how this important cichlid species detoxifies itself using enzymes [24]. Researchers have studied rhodanese in different animals, but they have not studied its biochemical characteristics in *C. zillii* before. This fills a huge gap in our understanding of fish enzymology and aquaculture biochemistry. *C. zillii* rhodanese has a molecular weight of 36 kDa, which is almost identical to the molecular weight of bovine liver rhodanese (33 kDa) [25] and human liver rhodanese (37 kDa) [26]. It shows that the structure of the enzyme has been preserved in all the vertebrate organisms. The molecular weight of the 17 kDa subunit, however, indicates a dimeric form, agreeing with some mammalian rhodaneses reported by Ogudugu et al. [27]. The optimum pH for *C. zillii* rhodanese is 8.0, which is slightly more basic than the optimum values for bovine rhodanese (pH 7.4) [28] and bacterial rhodanese from *Azotobacter vinelandii* (pH 7.8) [29]. This could be due to the fact that *C. zillii* occurs naturally in freshwater habitats with a higher pH value. The thermal stability profile indicates that *C. zillii* rhodanese remains completely active at 30 °C but rapidly loses its activity above 40 °C. This indicates that it is less thermally stable at high temperatures compared to thermophilic bacterial rhodaneses [30] but comparable in properties to other fish enzymes that have been adapted to temperate aquatic environments [31]. The substrate specificity analysis indicated a very clear order of preference: sodium thiosulfate (100%) > ammonium sulfate (80%) > mercaptoethanol (60%) > ammonium persulfate (30%). This concurs with the classical rhodanese mechanism, which favors the utilization of thiosulfate as the ideal sulfur donor [32]. The pattern of specificity is the same as that observed in mammalian rhodaneses but differs from other bacterial forms with greater tolerance of a wider substrate range [33]. The influence of salt on enzyme activity uncovered ionic needs unique to the species, with weak activation (25%) by KCl but other chloride salts (BaCl_2_, CaCl_2_, NaCl, MgCl_2_) exhibiting −12% to −28% inhibition [34]. The ionic sensitivity confirms that *C. zillii* rhodanese might have developed particular electrostatic surface contacts unique to the ionic status of freshwater habitats, unlike marine fish enzymes that are more salt tolerant [35]. The structure of the active site of *C. zillii* rhodanese was found by molecular docking experiments to have conserved catalytic residues typical of the conventional rhodanese mechanism. Good contacts of sodium thiosulfate with residues Glu159, Gln161, and Arg173 support the established two-step catalytic mechanism: sulfur transfer from thiosulfate to catalytic cysteine and sulfur transfer to the acceptor molecule [36]. What are the functions of the interacting residues Gln121 and Trp139? When ammonium sulfate is a substrate, such residues are likely to be functioning as secondary binding sites to orient the altered molecular geometry of sulfate with respect to thiosulfate [37]. Gln121 may contribute hydrogen bond stability to the oxygen atoms of the sulfate, and Trp139 could contribute orientation of the substrate by π-electron interactions, though with reduced affinity compared to the native thiosulfate substrate. The *C. zillii* rhodanese has vast possibilities in many industrial processes. In bioremediation, the enzyme is utilized for the detoxification of cyanide in the treatment of wastewater from mines, particularly its stability under moderate pH [38]. Pharmaceutical applications of this enzyme can be applied in drug metabolic studies and as a therapeutic reagent in the treatment of cyanide poisoning [39]. In addition, the substrate specificity pattern of the enzyme presents opportunities in analytical biochemistry to investigate sulfur compounds and in food science for the processing of sulfur compounds [40]. The intermediate thermostability of the enzyme and excellent activity under physiological pH conditions make it applicable to biotechnological uses with mild reaction conditions, perhaps with benefits over extremely thermostable but industrially problematic forms of the enzyme [41]. The occurrence of rhodanese in *C. zillii* would most likely be an adaptive survival mechanism against cyanogenic plant material or industrial contaminants in the environment [42]. Kinetic properties of the enzyme suggest optimization for this species’ freshwater environment, where cyanide can be derived from natural or man-made sources. Even with absolute biochemical characterization in this research, subsequent work should include crystallographic study to validate the postulated active site structure and vast kinetic studies with physiological acceptor compounds. Expression experiments under different environmental stress conditions could still highlight the enzyme’s implication in the *C. zillii* adaptation strategies.

## 5. Conclusions

The *C. zillii* rhodanese displays characteristic biochemical features that are unique compared to its mammalian and bacterial homologues but retains the inherent catalytic function. The enzyme is a valuable addition to the biotechnological repertoire and illuminates the evolutionary selection of detoxifying enzymes in freshwater fishes. The reported overall characterization provides the basis for future applications in bioremediation, analytical chemistry, and the pharmaceutical industries.

## Figures and Tables

**Table 1:** Purification Summary of Rhodanese from *C. zillii* Liver.

Purification Step	Volume (mL)	Total Protein (mg)	Total Activity (U)	Specific Activity (U/mg)	Yield (%)	Purification Fold
Crude Extract	75	401	690.00	1.72	100	1.00
70% Ammonium Sulphate	30	90	252.00	2.80	37	1.63
CM-Sephadex C-50	17	43	140.08	3.30	20	1.92
Biogel P-100	14	21	113.68	5.40	16	3.10

Values represent means of triplicate determinations. U = Unit of enzyme activity, defined as the amount of enzyme that catalyzes the formation of 1 μmol of product per minute under standard conditions.

**Table 2:** Docking Result Summary.

Substrate	Docking Score (kcal/mol)	H-Bond Donors	H-Bond Acceptors	Surface Interactions	Interacting Residues
Sodium thiosulfate	−4.372	Arg173-NH, Gln161-NH_2_	Glu159-COO, SO_4_ group	Strong donor-acceptor (magenta-green gradient)	Glu159, Gln161, Arg173, Arg156, Val172
Ammonium sulphate	−3.832	Arg156-NH, Gln121-NH_2_	SO_4_ group, Trp139-CO	Moderate donor-acceptor interface	Gln121, Trp139, Arg156
2-Mercaptoethanol	−2.853	Trp139-NH, Val172-NH	OH group, Val172-CO	Weak donor-acceptor surface contact	Gln121, Trp139, Val172
Ammonium persulphate	−1.488	Gln121-NH_2_	SO_4_ group	Limited surface complementarity	Gln121, Trp139

## Data Availability

The authors declare that the data supporting the findings of this study are available within the paper. Should any raw data files be needed in another format, they are available from the corresponding author upon reasonable request.
